# Supplementary material for: Fermentative α-Humulene Production from Homogenized Grass Clippings as a Growth Medium
Source: Molecules. 2022 Dec 8;27(24):8684. doi: 10.3390/molecules27248684 (PMC9788380; doi:10.3390/molecules27248684)
Supplement: Supplementary file 1 [file molecules-27-08684-s001.zip › molecules-2036034-SI.pdf]

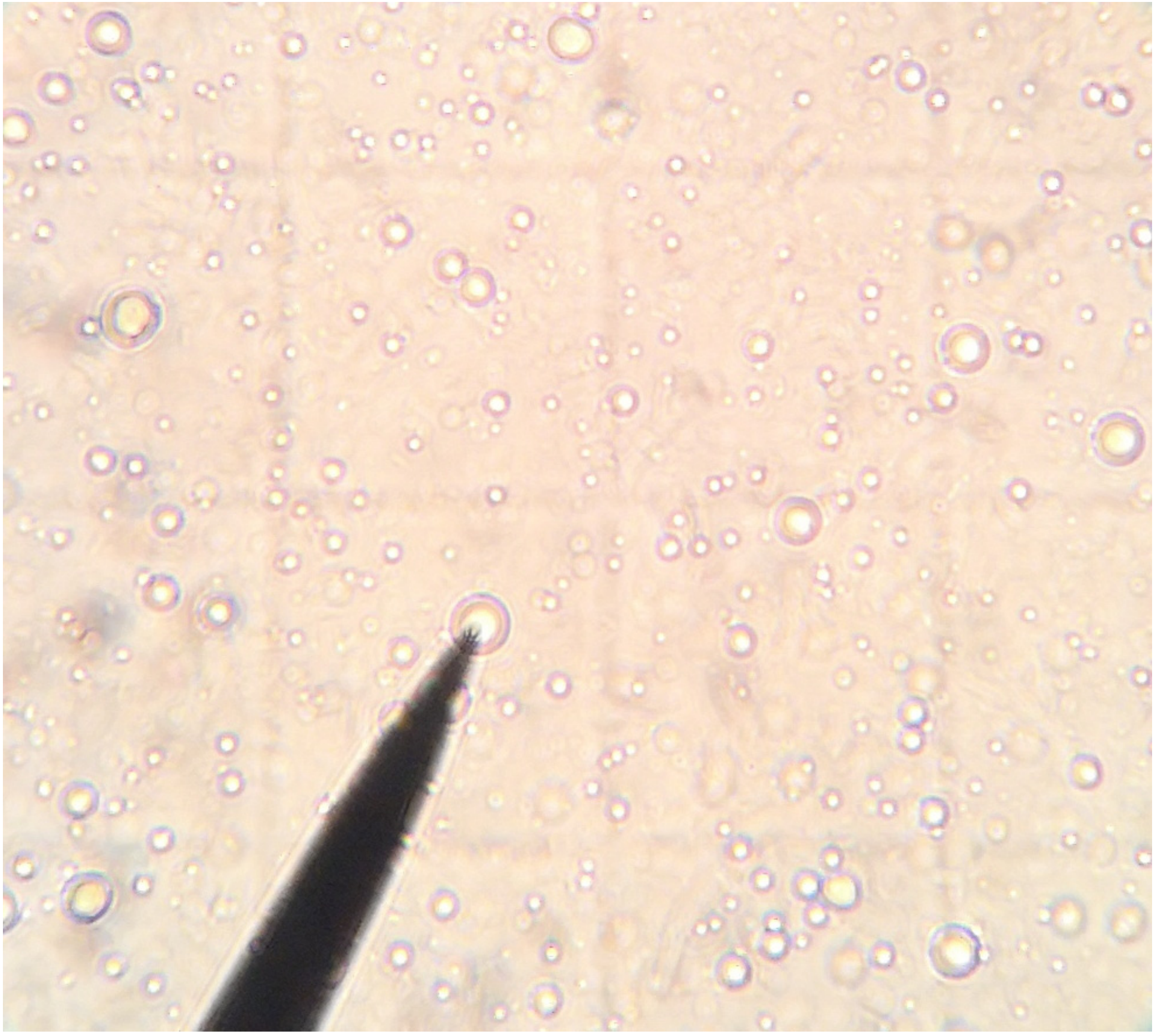

Figure S1: Microscopic image of a grass medium sample with dodecane after 48 h of cultivation (1000× magnification; squares have a size of 50 × 50 μm). The needle points to a vesicle of the oil-in-water emulsion of dodecane in the grass medium

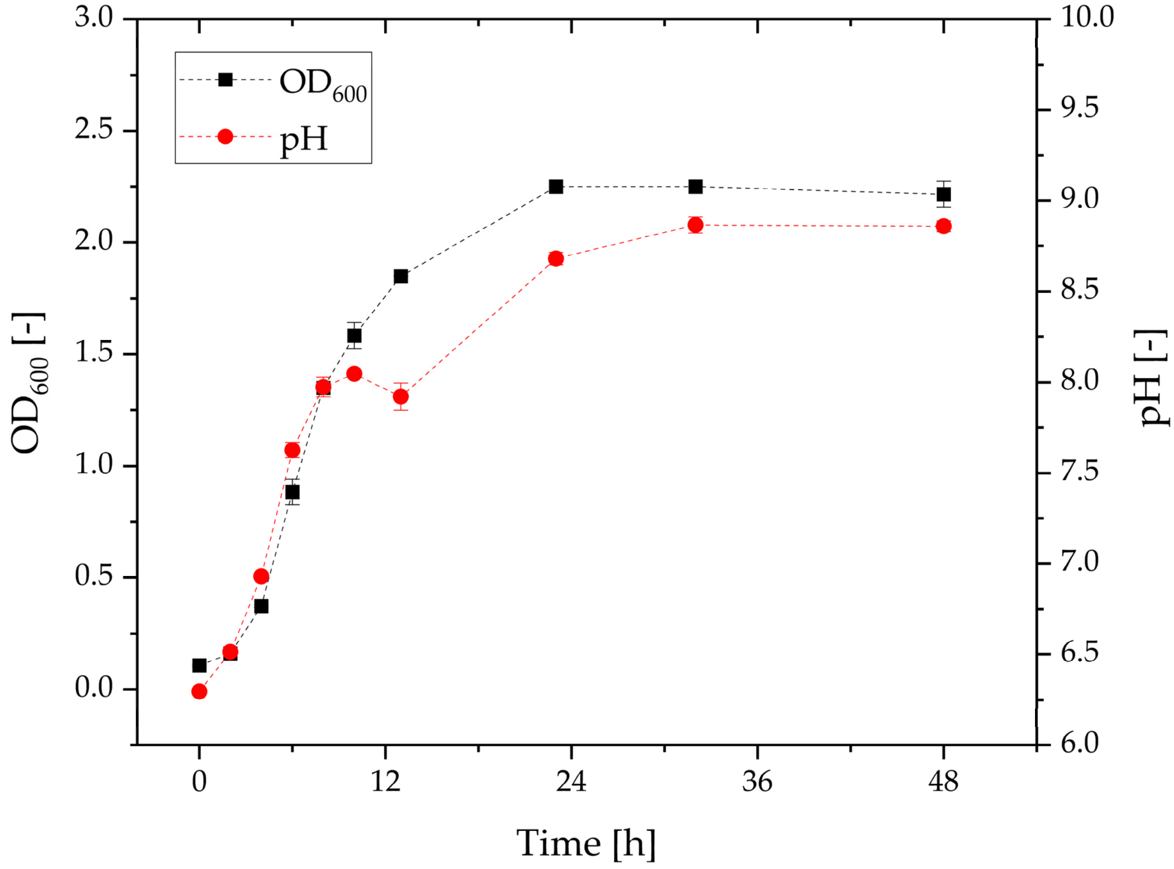

Figure S2: Growth curve and pH during the cultivation of *C. necator* pKR-hum in grass medium without the addition of dodecane (n = 3, dashed *line* serves as a *guide* to the *eye*).
